# The Young Interferometer as an Optical System for a Variable Depolarizer Characterization †

**DOI:** 10.3390/s19143037

**Published:** 2019-07-10

**Authors:** Aleksandra Kalbarczyk, Leszek R. Jaroszewicz, Noureddine Bennis, Monika Chrusciel, Pawel Marc

**Affiliations:** Faculty of Advanced Technologies and Chemistry, Military University of Technology, 00-908 Warsaw, Poland

**Keywords:** Young interferometer, depolarization measurement, modulation of depolarization, liquid crystal device

## Abstract

This article proposes an interferometric method for a variable depolarizer characterization with features that distinguish it from the polarimetric system. Information about the behavior of a vertically aligned nematic cell as a variable depolarizer can be extracted from Young interferometer measurements in real time. These results could be significant for understanding the polarization phenomena in depolarizing media such as biological tissue.

## 1. Introduction

Biological tissues are optically anisotropic. They exhibit birefringence because of their linear fibrous structure, which alters the polarization state of the incident light. Light transmitted or reflected by biological tissue may suffer from strong depolarization. This effect can be treated as a composition of two components of equal intensity but opposite state of polarizations (SOPs) such as two linear and perpendicular, left and right circular, or two general elliptical components with perpendicular azimuths and opposite helicity. These depolarization phenomena are associated with a reduction in the degree of polarization (DOP) of the incident beam. The value of the DOP of a monochromatic light beam is the ratio of the averaged intensity of the beam’s polarized portion to its total averaged intensity. An exact expression for DOP is included in the early work of G. G. Stokes [1], which was later presented by E. Wolf in terms of the electric field coherency matrix [2].

Therefore, the polarization properties of partially polarized light beams have traditionally been studied through the use of coherency matrices introduced in terms of the Stokes parameters. Random electromagnetic beams, which may have an arbitrary DOP, can be analyzed by interferometry [3] because interference is always observed, regardless of the nature of the light source and its SOP. The wavefield’s fundamental property to interfere was proposed by Fresnel and Arago [4].

As a matter of fact, two interfering beams’ polarizations play a very important role. If two beams are in orthogonally polarized states, no interference takes place, and the difference in polarization state reduces the visibility of interference fringes. However, the situation is quite different when the interference of two beams, which are completely time coherent and have the same intensity, is considered where one of the interacting beams is linearly polarized and the other is partially polarized. To determine the polarimetric signatures of biological tissues, several interferometric systems using unpolarized light have been developed in recent years [5]. This novel approach can be applied to low coherence interferometry, which has found an application in optical coherence tomography (OCT) to produce a two-dimensional image of optical scattering from internal tissue microstructures [6] and for medical imaging and industrial non-destructive testing (NDT) [7]. Finally, in a depolarizing instrument such as a broadband imaging spectrometer, depolarizers are placed in the system for optical signal stabilization. They are also used to reduce measurement offsets due to a strong polarization dependence. A dynamic depolarizer with a controllable DOP is also required to study the noise effect on quantum information.

However, to the best of our knowledge, no interferometric setup has been proposed to analyze the depolarized light in a time domain. In this paper, we report on the DOP investigation of light being transmitted through a variable depolarizer, while having the possibility of controlling the DOP with applied voltage. The results could be important for understanding polarization phenomena in the above-cited applications. The best known method of light depolarizing is light dispersion on porous structures [8]. In these cases, we can observe optical losses inherently associated with light scattering. To avoid losses, other light depolarization methods have been investigated. Many works have investigated liquid crystal (LC) depolarizing properties [9,10,11], but there are still many problems with this technology such as depolarizer instability with time or low depolarizing properties for sources with a high coherence length. In the present work, we propose a depolarizing LC material with specific alignment layers [12]. The most detailed description of a LC depolarizer is given by A. Shaham [13,14]. All successful designs have been based on his works in which the depolarizer scheme is composed of a sequence of birefringent crystals and wave plates. Wave plates were used instead of crystal direct rotation to eliminate an unwanted angle dependent retardation. These methods suffer from bulkiness and high cost. Accordingly, we incorporated the active element of a vertically aligned LC concept to create a beam with the desired DOP. Furthermore, this device had a time-varying birefringence. In our proposal, we used a modified Young’s interferometer to enable simple and automatic measurements of the time-varying phase with high sensitivity [15]. Our interferometer was adapted to include a half-wave plate (HP) and a quarter-wave plate (QP) in the interferometer’s object arm to generate an arbitrary polarization state in the input of the depolarizer cell, while the reference beam is kept linearly polarized.

In this extended paper with regard to the manuscript showed at the 7th International Symposium on Sensor Science (I3S 2019) [16], we present an interferometric optical system for a variable depolarizer characterization possessing the ability to study the optical phenomena in real time. In this paper, the manufacturing process of the LC depolarizer was optimized and an investigation conducted on the linearly polarized light in the reference beam. It should also be underlined that the measurement system concept was taken from [17], where the main difference is an implementation of a piezoelectric mirror adjuster in one arm of the interferometer as described in detail in Section 3. 

## 2. Liquid Crystal Variable Depolarizer

**Figure 1:**
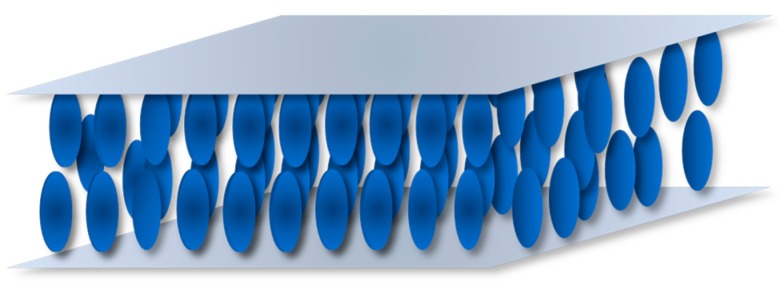
Homeotropic alignment in a nematic LC cell.

LCs are functional materials possessing anisotropies that originate from their inner molecular alignment. At a nematic substrate interface, as depicted in Figure 1, the tilt angle (α) is defined as the angle between the easy axis of the nematic molecules and the normal direction to the surface. Depending on the tilt angle, the alignment of the nematics can be categorized into two major groups: parallel (planar) or perpendicular (homeotropic) alignment. In the first one, the easy axis is parallel to the plane of the surface (the tilt angle α is equal to 90°), whereas in the second, the easy axis is vertical to the surface (α = 0°). A good approach for a disordered birefringence medium can be made upon switching OFF the vertically aligned nematic (VAN) liquid crystals. In the OFF state (voltage *V* = 0), it is isotropic for light impinging at a normal incidence. However, the electric field applied along the homeotropically aligned nematic LC with negative dielectric anisotropy makes the LC director start tilting away from the normal direction to the substrates. At Frederik’s transition, domains with lower birefringence start growing in the sample. The resulting optical switching, observed under crossed polarizers, shows many umbilical defects due to the molecules’ uncontrolled azimuthal reorientation. As a result, the device is capable of depolarizing coherent light beams [10,18]. The symmetrical construction of the cell assures that the performance will be the same for light coming from either side. 

**Figure 2:**
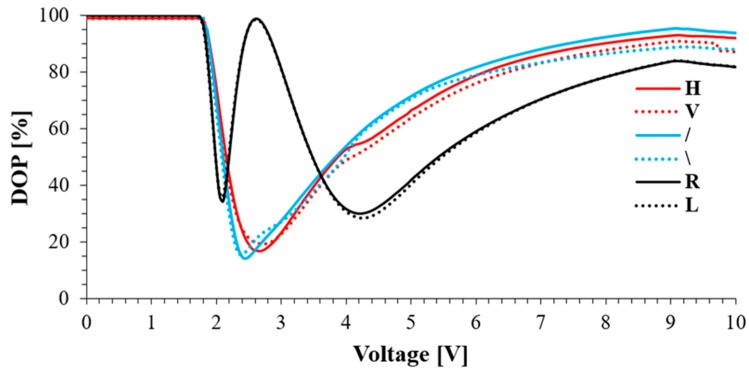
Measurement results of the DOP for VAN as a function of applied voltage for different input SOP; Linear horizontal (H) and vertical (V), linear at an angle of +45°(/) and −45°(\), circular right (R) and left (L).

For the proper validation of the depolarization properties of the VAN cell, it is highly desirable to measure the DOP that depends on the applied voltage and the SOP of the input beam:

(1) $$DOP=\frac{\sqrt{S_{1}^{2}+S_{2}^{2}+S_{3}^{2}}}{S_{0}^{2}},$$

where *S*_1_, *S*_2_, *S*_3_, *S*_0_ are Stokes parameters. In our experiment, a polarimeter (PAX5710VIS, Thorlabs) was employed to measure the DOP of the transmitted light. Figure 2 shows the measured DOP as a function of the applied voltage calculated from the experimentally measured Stokes parameters. Therefore, the DOP for VAN can be determined with high accuracy, as shown in Figure 2, for different inputs of SOPs [17]. These results show that with increasing applied voltage, the DOP of transmitted light decreases because the cell generates a disordered birefringent medium related to the undefined switching direction of molecules. This phenomenon produces mixed SOPs in the cross-section of the transmitted light. A series of the minima and maxima of the DOP are related to the cell’s retardation changes. For a linearly polarized beam, which passes through a depolarizer cell composed of a disordered birefringence medium, the DOP becomes zero when the optical retardation induced by the cell matches that of a half-wavelength of the incident beam. However, the incident circular polarization state DOP is zero when the phase retardation is tuned to the quarter-wave condition of incident circular polarization [19]. The proposed VAN cell transmits the polarized component of incident light with a minimum DOP (about 16%) for a horizontally polarized input light obtained at 2.65 V. In such situations, minimum intensity loss and scattering can be observed. It is also worth mentioning that the depolarizer cell’s orientation does not have an impact on the obtained measurements.

## 3. Calibration and Essential Parameters of the Applied Interferometric Measurement Setup

**Figure 3:**
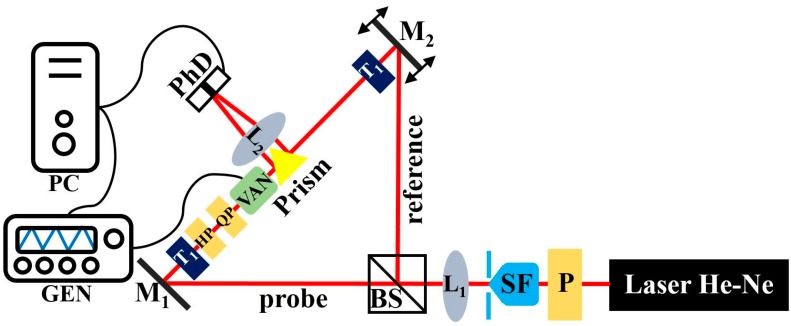
The scheme of the interferometer setup. Light source: He-Ne laser with λ = 633 nm; P—linear polarizer; SF—spatial filter; L_1_, L_2_—lenses, BS—beam splitter; M_1_, M_2_—mirrors; T_1_, T_2_—telescopes, VAN—vertically aligned nematic liquid crystal cell; PhD—photodetector, Gen—generator, HP—half-wave plate, QP—quarter-wave plate. Adapted from [16,17].

The adequate tool for studying the dynamic effects of the spatial distribution of birefringence upon switching a VAN cell by an electric field was a modified Young’s interferometer proposed in our previous works [15,16,17]. This interferometer was constructed in free space with the possibility of controlling the fringe pattern in real time to study polarization dynamics and their fluctuations in the time domain. This optical setup is conceptually simple and is analogous to the two-slit experiment in many ways. The diffraction field is a result of the interference of light beams coming out from the interferometer’s two arms with a controlled beam diameter. The signal from one arm is a reference signal relative to which the phase difference in the other arm will be determined. This phase difference will affect the shifting of the fringes in the resulting interference field. The proposed interferometric system is presented in Figure 3. The light from a He–Ne laser (λ = 633 nm) passes through the polarizer (P) and is spatially filtered (SF) and collimated by the lens (L_1_). Next, the light splits into two beams by the beam splitter (BS). The probe beam is reflected by the mirror (M_1_), and a reference beam is reflected by the second mirror (M_2_), which is mounted on the mirror mount with the piezoelectric adjuster. Changing the optical path in one arm of the interferometer causes the phase shift change. In both paths, telescopes (T_1_, T_2_) consisting of two positive lenses were inserted. To adjust the reference equality and the probe beams’ diameter, two collimators with a circular pinhole were situated after the telescopes to obtain 400 μm diameter collimated beams (*d* = 400 µm). The interferometer includes a half-wave plate (HP) and a quarter-wave plate (QP) in the probe arm to change the beam incident polarization, while the reference beam is kept linearly polarized. Then, a right-angled gold coated prism bends the light coming from the collimators by 90°. Thus, the reflected beams become parallel to each other and are focused by the Fourier lens (L_2_, *f* = 170 mm) on a two-channel photodetector (PhD). The PhD consists of the right (PhD_R_) and left (PhD_L_) photodetectors separated by a gap (*q* ≈ 50 μm). The prism is mounted on a translation and rotation stage. The possibility of setting the prism position enables us to change the distance between two beams (*2b*) and the distribution of the fringe pattern, i.e., the number of fringes. These two parameters, *2b* and *q*, significantly affect our setup performance. Due to the fact that *q* is fixed, the calibration process requires the adjustment of *2b*, which is affected by the prism position. If the prism position is changed, the distribution of the field intensity in the Fourier plane collected by PhDs also changes. These intensities are collected in an acquisition card (DAC). Software for data recording was created in LabView. 

**Figure 4:**
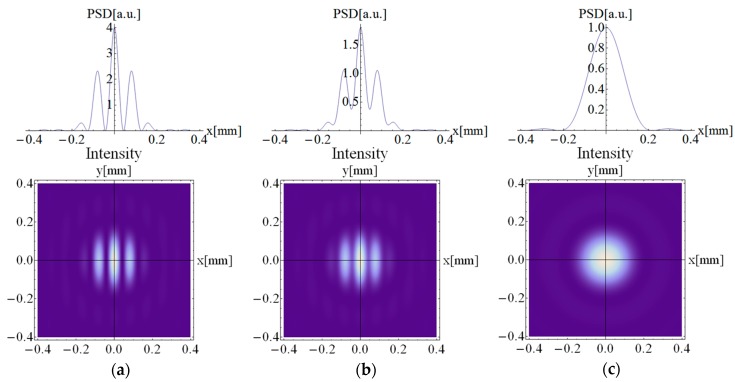
Simulation results of the distribution of the power spectral density (PSD) and the corresponding interferograms of the fringe patterns for a different polarization orientation difference (θ) between the interfering beams: (**a**) θ = 0°, (**b**) θ = 70°, and (**c**) θ = 90°.

Interference between two beams with the same linear polarization that emerges from two single mode optical fibers can be analyzed by means of a bicell photodetector as used in [15]. In the present work, it is intended that an equivalent experimental configuration was appropriate to study the phase shift when the polarization state between two beams is different. The reference beam has a linear polarization while the probe beam has any possible polarization state. Consequently, a theoretical model has been built up describing the effect of the polarization orientation difference (θ) between the interfering beams. In this case, the amplitude transmittance of the circular apertures is given by:

(2) $$f\left(r\right)=circ\left(\frac{r-b}{d}\right)e^{i\varphi_{2}}+\cos\left(\theta \right)circ\left(\frac{r+b}{d}\right)e^{i\varphi_{1}},$$

where $\varphi_{1},\varphi_{2}$ is the phase in the probe and reference arm of the interferometer, respectively, and $r=\sqrt{x_{1}^{2}+y_{1}^{2}}$ are coordinates in the Fourier objective plane. The final form describing the relationship between the intensity distribution with respect to the fringes contrast is [20]:

(3) $$I={\left(\frac{\pi d}{f\lambda }\right)}^{2}{\left(\frac{2J_{i}\left(\pi d\rho \right)}{\pi d\rho }\right)}^{2}(1+\cos^{2}\left(\theta \right))\left[1-\mu \cos\left(4\pi bu-\Delta \varphi \right)\right],$$

where *J_i_* is the Bessel functions; $u=\frac{x}{\lambda f}$, $v=\frac{y}{\lambda f}$ are the components of the spatial frequencies in the x and y directions in the Fourier plane; $\rho =\sqrt{u^{2}+v^{2}}$; and $\mu =\frac{2\cos\left(\theta \right)}{1+\cos^{2}\left(\theta \right)}$. Equation (3) is the basic dependency on the basis of which a number of simulations have been made. The simulations of the cross-section of power spectral density (PSD) and the corresponding interferograms of the fringe patterns for three different values of θ (θ = 0°, θ = 70°, and θ = 90°) are presented in Figure 4. As can be seen, the PSD envelope is the same for all cases because it depends on the beam’s aperture d. Along with the θ change, the PSD distribution is averaged until the disappearance of the fringes at θ = 90°.

**Figure 5:**
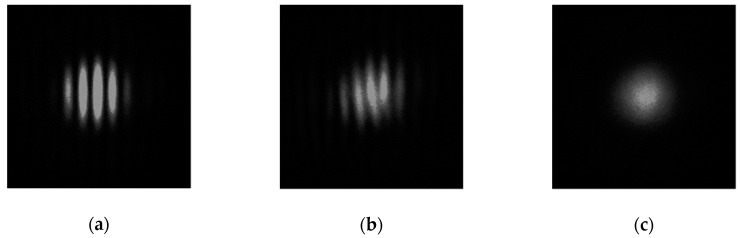
The fringe pattern photos captured by the CCD camera placed in the Fourier plane. The VAN cell was inserted in the interferometer’s probe arm. Linear horizontal polarization of the incident beam was used and the reference beam was linearly polarized. By applying different voltages to the cell, the DOP of the probe beam was: (**a**) 100%, (**b**) 50%, and (**c**) 2%.

The actual shapes of the fringe patterns captured by the CCD camera (The Imaging Source, DMK 41BF02) that were inserted in the Fourier plane in place of the PhD (from Figure 3) are presented in Figure 5. The pictures were taken while the reference and probe arm were horizontally polarized, and the VAN cell was inserted in the probe arm. In Figure 5a, the interference field showed high contrast and high uniformity of distribution. In this case, the voltage was not applied to the LC, therefore no depolarization could be observed (DOP = 100%), and the resulting fringe pattern corresponded to the simulated image presented in Figure 4a. However, with increasing voltage, the molecules started to switch in arbitrary directions, causing the depolarization effect. In Figure 5b, the fringe pattern picture was taken when the VAN cell was switched by voltage. In this situation, the DOP value in the probe arm was about 50% for an applied voltage of 2 V. As can be seen, the contrast in the fringes decreased. Figure 5c shows the situation when the DOP reaches the minimal value (DOP = 2%) at 3.1 V. In this case, no interference pattern can be observed. It is worth emphasizing that as long as the size of the incident beam affects the performance of the depolarization capability of the VAN cell, the correspondence between the voltage values and DOP obtained from the interferometric measurements (beam size = 400 µm) is not comparable with the polarimetric measurements shown in Figure 2 (beam size about 4 mm). Nevertheless, the apparent asymmetry and deformation in the interference pattern detected in Figure 5 may be due to the depolarization of the object beam. Hence, the intensity distribution presented in Figure 5c is not equivalent to the situation shown in Figure 4c, where the interference field of two orthogonal beams is presented.

The main goal of this paper was to measure the phase shift caused by the VAN cell switching. The phase shift of the fringes can be observed as the DOP in liquid crystals is associated with the retardation as described in [18]. Detected signals from PhD_R_ (*S_R_*) and PhD_L_ (*S_L_*) can be described as follows:

(4) $$S_{R}=a_{1}+a_{2}\cos\Delta \varphi -a_{3}\sin\Delta \varphi ,$$

(5) $$S_{L}=a_{1}+a_{2}\cos\Delta \varphi +a_{3}\sin\Delta \varphi .$$

where Δφ is the phase shift and *a*_1_, *a*_2_, *a*_3_ are the Fourier coefficients. Then, using the relationship describing signals from the left and right side, the sum *Sum* = (*S_R_* + *S_L_*) and the difference *Diff* = (*S_R_* − *S_L_*) can be expressed as:

(6) $$Sum\left(\Delta \varphi \right)=2a_{1}+2a_{2}\cos\Delta \varphi ,$$

(7) $$Diff\left(\Delta \varphi \right)=2a_{3}\sin\Delta \varphi .$$

According to Equations (6) and (7), *a*_1_ is the amplitude of the sum function, *a*_2_ is the average value of the sum function, and *a*_3_ is the average value of the difference function. From simple trigonometry, tan(Δφ)=sin(Δφ)/cos(Δφ), the phase difference can be expressed as:

(8) $$\tan\left(\Delta \varphi \right)=\frac{Diff\left(\Delta \varphi \right)a_{2}}{a3\left(Sum\left(\Delta \varphi \right)-2a_{1}\right)}.$$

One can see that *S_R_* and *S*_L_ depend on the combination of cosine and sine of the measured phase shift. The coefficients *a*_1_, *a*_2_, *a*_3_ are constants depending on the wavelength *λ*, the distance between two beams *2b*, the focal length *f* of the Fourier lens, the polarization, and the size of the beam *d*. The validation of *a*_1_, *a*_2_, *a*_3_ is essential to optimize the system operation. Constants *a*_1_, *a*_2_, *a*_3_ can be determined starting from a dependence describing the intensity:

(9) $$I\left(x\right)={\left|E\left(x\right)\right|}^{2}={\left|E_{1}exp\left(i\frac{\Delta \varphi }{2}\right)+E_{2}^{*}exp\left(-i\frac{\Delta \varphi }{2}\right)\right|}^{2},$$

where

(10) $$E_{1}=\exp\left(i2\pi bu\right)\left(\frac{2J_{1}\left(\pi d\rho \right)}{\pi d\rho }\right),$$

(11) $$E_{2}^{*}=\cos\left(\theta \right)\exp\left(-i2\pi bu\right)\left(\frac{2J_{1}\left(\pi d\rho \right)}{\pi d\rho }\right),$$

(12) $$A^{2}\left(\rho \right)=\left(\frac{2J_{1}\left(\pi d\rho \right)}{\pi d\rho }\right).$$

*A*^2^(*ρ*) is the spatial distribution of amplitude in the Fourier plane and $J_{1}$ is the Bessel function of the first-order. The above formulas are components which take into account the form of the intensity distribution after Fourier transform with the polarization orientation difference *θ* between the interfering beams. Then, expressing the above components of complex functions in the form of trigonometric functions, the intensity distribution can be described as:

(13) $$I\left(x\right)=A^{2}\left(\rho \right)\left\{1+\cos^{2}\left(\theta \right)+\cos\left(\theta \right)\left[2\cos\left(4\pi bu\right)\cos(\Delta \varphi )-2\sin\left(4\pi bu\right)\sin\left(\Delta \varphi \right)\right]\right\}.$$

**Figure 6:**
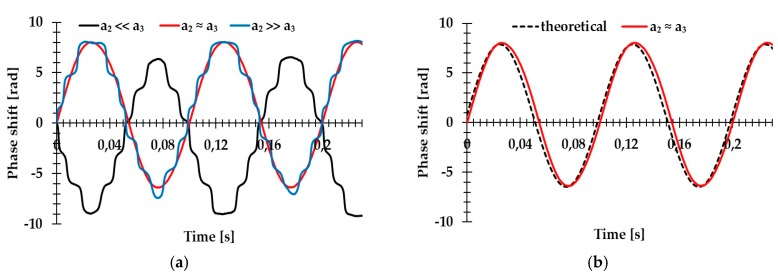
System calibration. (**a**) Dynamic phase shift measurements at three different values of the distance between two beams *d.* (**b**) Comparison of the theoretical model (dotted line) and measured phase shift when coefficients *a*_2_ and *a*_3_ are equal. Adapted from [16].

According to Equations (9)–(11), the integration coefficients of the cross-section of the intensity distribution can be determined. The integration concerns the selected cross-section in the *x* direction:

(14) $$a_{1}=const\int_{q/2}^{\infty }A^{2}\left(\rho \right)\left[1+\cos^{2}\left(\theta \right)\right]dx=const\int_{-\infty }^{-q/2}A^{2}\left(\rho \right)\left[1+\cos^{2}\left(\theta \right)\right]dx,$$

(15) $$a_{2}=const\int_{q/2}^{\infty }A^{2}\left(\rho \right)\left[2\cos^{2}\left(\theta \right)\cos\left(4\pi bu\right)\right]dx=const\int_{-\infty }^{-q/2}A^{2}\left(\rho \right)\left[2\cos^{2}\left(\theta \right)\cos\left(4\pi bu\right)\right]dx,$$

(16) $$a_{3}=const\int_{q/2}^{\infty }A^{2}\left(\rho \right)\left[2\cos^{2}\left(\theta \right)\sin\left(4\pi bu\right)\right]dx=-const\int_{-\infty }^{-q/2}A^{2}\left(\rho \right)\left[2\cos^{2}\left(\theta \right)\sin\left(4\pi bu\right)\right]dx,$$

where *q*/2 is half of the distance between two PhDs. As above-mentioned, the ratio of the coefficients *a*_2_ and *a*_3_ is influential. The experimental investigations of coefficients *a*_2_, *a*_3_, and the calibration of the setup were performed using a mirror (M_2_) mounted on a moving table with a piezoelectric adjuster. Thus, the phase shift was induced in our system. During the calibration process, the mount of the mirror was moved by the external electric field. Results of the phase demodulation as a function of time for three different positions of the prism are presented in Figure 6a. As can be seen, the variation of the distance between two beams had a significant impact on the values of coefficients *a*_2_ and *a*_3_ as well as on the performance quality of our device. If one of the coefficients was much larger, the resulting phase shift recorded by our system was not linear. This error had to be eliminated during the measurement. In the case where *a*_2_ ≈ *a*_3_, the experimental results were in good agreement with the theoretical model presented in Figure 6b. The theoretical model was calculated from Equation (8), assuming the optical path shift induced by the mirror displacement.

## 4. Analysis of Dynamic Polarization Transmitted from the VAN Cell

**Figure 7:**
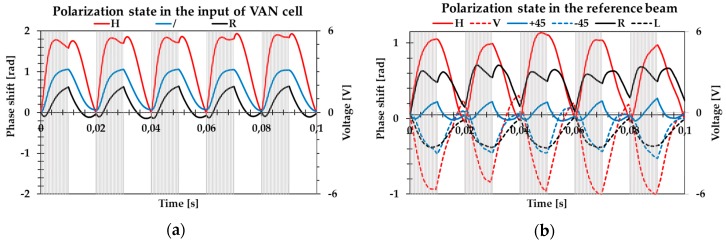
Real time phase shift measurements when the modulated waveform varied between zero and 6 V was applied to the vertically aligned nematic (VAN) cell. (**a**) Arbitrary polarization state in the input of the depolarizer while the reference beam is kept linearly polarized. (**b**) Horizontal polarization input while the reference beam polarization has been changed to horizontal (H), vertical (V), linear ±45°(+45 and −45), circular left (L), and circular right (R).

As a result of the LC’s electro-optical tunability, the proposed depolarizer could be electrically tunable. Therefore, we could switch the cell between OFF (non-depolarizing) and ON (depolarizing) states. The waveform applied to the VAN was a 1 kHz AC voltage modulated by a 50 Hz envelope with a 6 V amplitude. The beams in both arms were adjusted in such a way that their intensities were equal on recombination. The recombined beam showed interference fringes that resulted from geometrical path differences between the two arms. Figure 7a shows the phase shift measurements in the case where the arbitrary polarization state was selected at the input of the VAN cell while the reference beam was kept linearly polarized. In the three cases of incident polarization, the DOP switched from 100% in the OFF state to nearly 10%, 32%, and 45% when the incident beam was linear horizontal, +45°, and a circular right polarization light beam, respectively. For a linear horizontal polarization of the incident beam, it could be seen that the phase shift stabilized at the value of 1.8 radians, while in the case of the +45° polarization, the phase shift stabilized at 1.0 radian, and with the circular polarization of the incident beam, the phase shift stabilized at about 0.5 radian. Different values of phase modulation depth were associated with the depolarization capability of the device for different SOPs of the incident beam.

These results show that when two light beams have perfect temporal coherence and the same intensity interferes, but one beam has a linear polarization while the other is partially polarized, the interference occurs. The DOP value of a depolarized beam is associated with a phase shift of the interfering beams in such a way that the minimum DOP is produced when the optical retardance is at its maximum. In the particular case where the linear horizontal polarization of the incident beam is used and the reference beam is linearly polarized, temporal modulation of the controlled voltage applied to the VAN cell produces temporal phase-shifted interferograms corresponding to ON and OFF states. The VAN cell in the OFF state has a homeotropic order. As a consequence, the cell is isotropic for light impinging at normal incidence, whereby a non-phase shift is obtained between the two interfering beams. However, when the voltage is ON, the electric field makes the orientation of the molecules of the LC undefined, resulting in the spatial distribution of birefringence, which depolarizes the light emerging from the VAN cell at the interference field. In this case, the superposition of a randomly polarized object beam with a horizontally polarized reference beam results in a fringe pattern with a reduced contrast. The rise time of molecular reorientation from homeotropic alignment (zero phase shift) to disordered reorientation (maximum phase shift) can be measured from the phase modulation response which is about 4 ms. After the electric field is removed, molecular relaxation is back to a well-defined homeotropic state with a relaxation time of about 7 ms. During the pulse duration, numerous umbilical defects induced by the applied electric field to the nematic liquid crystal are generated. These defects have a dynamic instability due to annihilation over time [21]. The characteristic fluctuation of the phase during pulse duration may give information about the dynamic annihilation of the umbilical defect.

The fact that the phase shift depended on the object beam’s DOP in our experiment, we conducted an interferometric analysis for the spatial distribution of varying polarization in a cross-section of the depolarized beam transmitted by the VAN cell.

Figure 7b shows the results of the phase shift measurements when the horizontal polarization state illuminated the depolarizer, while in the reference beam, the arbitrary polarization state was generated. The measurements show that the modulation depth was comparable with opposite SOPs, i.e., horizontal and vertical or +45° and −45° of the reference probe beam. However, the sign of the phase shift was different, which indicates that the two orthogonal components of the depolarized beam electric field are guided in opposite optical paths. This result reflects the correlation between the orthogonal equal-intensity electric field components at a single space-time point, which can be considered as a degree of correlation between the orthogonal components, which is a fundamental definition of unpolarized light.

This method, implemented to investigate the polarization properties of the depolarized beam, could give us new information about the tested depolarizer that cannot be observed by standard methods. However, the proper treatment of the problem involving depolarized beams in the interferometer does not appear as obvious as it should be. Our real time analysis establishes a new interferometric interpretation for the DOP of an unpolarized beam, which will be the object of further developed study.

## 5. Conclusions

This article presented a method of registration and interpretation of the beam degree of polarization (DOP) by applying the measurement of the phase shift recorded in the Young interferometer system. The recorded dynamic phase shifts were related to the DOP of the beam obtained in the electro-optical depolarizer using phase modulation for depolarization. The presented method allowed us to extract information on the dynamic effects related to spatial birefringence distribution in vertically ordered nematic (VAN) liquid crystal cells switched by means of an electric field. These effects cause a homogeneous spatial birefringence distribution in the cell, thus the light coming out of the VAN becomes depolarized.

This proposed study could find future application in sensing devices as well as in studies on light depolarization transmitted or reflected by different types of depolarizing media.
